# Characterization and antioxidant activity of nano-formulated berberine and cyperus rotundus extracts with anti-inflammatory effects in mastitis-induced rats

**DOI:** 10.1038/s41598-024-66801-8

**Published:** 2024-08-09

**Authors:** Aml E. Hashem, Ingi H. Elmasry, Mohamed A. Lebda, Dina R. S. Gad El-Karim, Mohamed Hagar, Sawsan Kh. M. Ebied, Badriyah S. Alotaibi, Nermin I. Rizk, Heba I. Ghamry, Mustafa Shukry, Hanan A. Edres

**Affiliations:** 1 Department of Biochemistry, Faculty of Veterinary Medicine, Alexandria University, Alexandria, Egypt; 2 Department of Pathology and Clinical Pathology, Faculty of Veterinary Medicine, Alexandria University, Alexandria, Egypt; 3 Department of Chemistry, Faculty of Science, Alexandria University, Alexandria, Egypt; 4 Faculty of Advanced Basic Sciences, Alamein International University, Alamein City, Matrouh Governorate Egypt; 5 Bacteriology Unit, Animal Health Research Institute, Alexandria Province, Egypt; 6 Department of Pharmaceutical Sciences, College of Pharmacy, Princess Nourah bint Abdulrahman University, P.O. Box 84428, Riyadh, 11671 Saudi Arabia; 7 Medical Physiology Department, Faculty of Medicine, Menoufia University, Menoufia, Egypt; 8 Nutrition and Food Science, Department of Biology, College of Science, King Khalid University, P.O. Box 960, Abha, 61421 Saudi Arabia; 9 Department of Physiology, Faculty of Veterinary Medicine, Kafrelsheikh University, Kafrelsheikh, 33516 Egypt

**Keywords:** Mastitis, Berberine, *Cyperus rotundus*, NPs, Characterization, Biochemistry, Health occupations

## Abstract

Bovine mastitis caused by infectious pathogens, mainly *Staphylococcus aureus* (*S. aureus*) and *Escherichia coli* (*E. coli*), constitutes a major destructive challenge for the dairy industry and public health. Berberine chloride (BER) and *Cyperus rotundus* possess a broad spectrum of anti-inflammatory, antioxidant, antibacterial, and antiproliferative activities; however, their bioavailability is low. This research aimed first to prepare an ethanolic extract of *Cyperus rotundus* rhizomes (CRE) followed by screening its phytochemical contents, then synthesis of BER and CRE loaded chitosan nanoparticles (NPs) (BER/CH-NPs and CRE/CH-NPs), afterward, the analysis of their loading efficiency in addition to the morphological and physicochemical characterization of the formulated NPs employing Scanning Electron Microscopy (SEM), Zeta Potential (ZP), Fourier Transform Infrared Spectroscopy (FTIR), Differential Scanning Calorimetry (DSC) and X-Ray Diffraction (XRD) assessments compared to their crude forms to evaluate the enhancement of bioavailability and stability. Isolation of bacterial strains from the milk of mastitic cows, used for induction of mammary gland (MG) inflammation in female albino rats, and a preliminary investigation of the prophylactic oral doses of the prepared NPs against *S. aureus*-induced mastitis in female rats. The minimal inhibitory concentration (MIC) of BER/CH-NPs and CRE/CH-NPs is 1 mg/kg b.w. BER/CH-NPs and CRE/CH-NPs alone or in combination show significant (P ≤ 0.05) DPPH radical scavenging activity (69.2, 88.5, and 98.2%, respectively) in vitro. Oral administration of BER/CH-NPs and CRE/CH-NPs to mastitis rats significantly (P ≤ 0.05) attenuated TNF-α (22.1, 28.6 pg/ml), IL-6 (33.4, 42.9 pg/ml), IL-18 (21.7, 34.7 pg/ml), IL-4 (432.9, 421.6 pg/ml), and MPO (87.1, 89.3 pg/ml) compared to mastitis group alongside the improvement of MG histopathological findings without any side effect on renal and hepatic functions. Despite promising results with BER and CRE nanoparticles, the study is limited by small-scale trials, a focus on acute administration, and partially explored nanoparticle-biological interactions, with no economic or scalability assessments. Future research should address these limitations by expanding trial scopes, exploring interactions further, extending study durations, and assessing economic and practical scalability. Field trials and regulatory compliance are also necessary to ensure practical application and safety in the dairy industry. In conclusion, the in vitro and in vivo results proved the antioxidant and anti-inflammatory efficacy of BER/CH-NPs and CRE/CH-NPs in low doses with minimal damage to the liver and kidney functions, supposing their promising uses in mastitis treatment.

## Introduction

In the last decades, phytochemicals carried significant concerns due to their biological compounds' merits not to induce bacterial resistance in contrast to antibiotics^1^. Furthermore, drug delivery in association with NPs expressed multiple profits regarding the improvement of drug distribution, enhancement of solubility, bioavailability, targeting, and controlled drug release^2^. Berberine (BER) is a quaternary amine found in many plants like *Ranunculacae*, *Berberidaceae* and *Rutacea*; rich in isoquinoline alkaloid and possesses anti-inflammatory, immunomodulatory, hypolipidaemic, hypoglycemic, anti-neoplastic, hepatoprotective^3^, antibacterial, antifungal, antiviral and antiparasitic activities; but with poor intestinal absorption, rapid metabolism, and low bioavailability when administrated orally^4^. *Cyperus rotundus* (CRE, Nut Sedge) is another herbal plant whose extract exerts anti-inflammatory, anti-cancer, immunomodulatory^5^, analgesic, antibacterial, and antioxidant properties as it comprises plenty of chemical compounds like alkaloids, flavonoids, glycosides, monoterpenes, furochromones, glycerol and tannin^5^. It is a traditional medicinal plant in the Cyperaceae family that grows in tropical and subtropical countries^6^. The advantages of phytochemicals over antibiotics in terms of bacterial resistance and the benefits of nanocarriers in enhancing drug bioavailability and targeting. For instance the, berberine effectively inhibits multi-drug resistant bacteria like MRSA by disrupting cell membranes and inhibiting efflux pumps^7^. Mun et al.^8^ highlighted curcumin's broad-spectrum antibacterial activity with a low tendency to induce resistance. Thymol and carvacrol which are effective against antibiotic-resistant bacteria by disrupting membranes and biofilm formation^9^. Additionally, curcumin-loaded nanoparticles significantly improved bioavailability and therapeutic efficacy in cancer treatment^10^. Berberine-loaded liposomes increased bioavailability and antibacterial activity through sustained release and targeted delivery^11^. Thymol-loaded chitosan nanoparticles observe enhanced antibacterial activity and reduced cytotoxicity^12^. The antimicrobial properties of various plant-derived compounds emphasize their effectiveness against resistant bacterial strains^13^. Resveratrol-loaded nanoparticles improved bioavailability and antioxidant activity, highlighting the potential for treating oxidative stress-related diseases^14^. These examples underscore the potential of phytochemicals and nanotechnology in overcoming limitations in antibiotic therapy, offering new avenues for combating bacterial resistance and improving drug delivery systems.

Most flavonoids, polyphenols, and phenolic acids are hydrophobic with minimal bioavailability, which reduces their oral therapeutic potency, as they are poorly absorbed from the gastrointestinal tract and rapidly metabolized; hence, to overcome this hindrance, a drug delivery system (DDS) could be applied to Permit the delivery and release of medicinal agents to enhance their efficiency^15^. Nanocarriers are one of the essential DDSs, which control the pharmacodynamics and pharmacokinetics of drugs, permitting the delivery of active molecules to the desired target site on account of their surface modification capacity and small size, which assist drug absorption and distribution to the target tissue as well as augmenting the drug solubility and bioavailability and diminishing its side effect^16^. Chitosan is one of the most promising nanocarriers^17^ as it can enhance the intestinal absorption of the loaded drug^18^ in addition to its advantages as an antimicrobial, antioxidant, and immunomodulatory agent. It is derived from chitin, which is the primary component of the cell walls of various aquatic and microorganisms^19^.

This study introduces several innovative elements in treating bovine mastitis, leveraging nanotechnology to enhance the therapeutic effects of natural compounds berberine chloride (BER) and *Cyperus rotundus* extract (CRE). By encapsulating these compounds in chitosan NPs, the research aims to overcome their naturally low bioavailability, enhancing solubility and stability. The literature concerning the effect of CRE-NPs against mastitis is sparse; thus, this study also aims to compare the efficacy of CRE and BER-NPs in a bacterial-induced mastitis model in rats. The NPs are thoroughly characterized using advanced techniques such as SEM, ZP, FTIR, DSC, and XRD, ensuring optimal design for therapeutic delivery. Additionally, the study investigates the minimal inhibitory concentration (MIC), antioxidant capacity, and anti-inflammatory activity of both CRE and BER-NPs. This comprehensive approach extends beyond formulation to evaluate the prophylactic and therapeutic efficacy in a rat mastitis model alongside a safety assessment focusing on renal and hepatic functions. This multifaceted approach promises to improve mastitis management in dairy cattle and represents a significant advancement in the application of nanotechnology in veterinary medicine.

## Materials and methods

### Ethical approval

The international ethical guideline for caring for and handling laboratory animals for rats was performed. Faculty of Veterinary Medicine, Alexandria University, Egypt, Institutional Animal Care and Use Committee (Approval No. AU-IACUC 013-22-12-12-(3) 176) approved the experimental procedure, confirming that all experiments followed relevant guidelines and regulations. This work was carried out following ARRIVE guidelines (https://arriveguidelines.org).

### Chemicals

Berberine chloride, Chitosan, and Dimethyl sulfoxide (DMSO) were purchased from SIGMA- ALDRICH, Co. Sodium tripolyphosphate anhydrous (TPP) from LOBA CHEMIE PVT.LTD. Acetic acid glacial 99% from Fisher Scientific, UK. Mannitol salt agar, MacConkey agar, eosin methylene agar, blood agar base, and Tripticase soya broth were purchased from Oxoid. Tumor Necrosis Factor-alpha (TNF-α), Interleukin-4 (IL-4), Interleukin-6 (IL-6), Interleukin-18 (IL-18), and Myeloperoxidase (MPO) were obtained from Abcam (USA), and Kits for creatinine and ALT were obtained from Vitro Scient. Egypt.

### *Cyperus rotundus* extract preparation and phytochemical screening

Our experimental and field research on plants, whether cultivated or wild, adheres to applicable institutional, national of Alexandria University, and international guidelines and legislation regarding the collection of plant material. We have ensured that all necessary permissions and compliance measures have been taken following these standards.

The rhizomes were cut into small pieces and ground into powder. This rhizome powder was then dissolved in absolute ethanol at a ratio of 1:10 (drug: solvent, w/v) inside an aluminum-covered flask to protect it from sunlight. The mixture was agitated using a magnetic stirrer. Subsequently, the solution was filtered to remove any impurities, and the solvent was evaporated using a rotary evaporator at 1000 rpm for 24 h at a temperature of 70 °C. This process aimed to maintain the closest resemblance to the form of the plant used by the general public, who typically obtain it from herbalists^5^.

Phenolic and flavonoid compound analyses were conducted following the methods described by Lin et al.^20^ and Kuntic et al.^21^ using an Agilent Series 1100 HPLC apparatus. The HPLC system consisted of an auto-sampling injector, solvent degasser, two LC pumps, Chem Station software, and a UV/Vis detector set at 250 nm for phenolic acids and 360 nm for flavonoids. A C18 column (125 mm × 4.60 mm, 5 µm particle size) was used for separation.

For the separation of phenolic compounds, a gradient mobile phase was employed, consisting of methanol (Solvent A) and acetic acid in water (1:25) (Solvent B). The gradient program started with 100% Solvent B for the first 3 min, followed by 50% eluent A for the next 5 min. The concentration of Solvent A was then increased to 80% for 2 min and reduced back to 50% for the following 5 min. Detection was performed at 250 nm. The separation of flavonoids was achieved using an isocratic elution program with a mobile phase composed of acetonitrile (A) and 0.2% aqueous formic acid (B) in a ratio of 70:30. The solvent flow rate was 1 ml/min, and the separation temperature was maintained at 25 °C. The injection volume was 25 μl. The total phenolic content of the *Cyperus rotundus* extract (CRE) was determined using a colorimetric method with Folin-Ciocalteu reagent, following the protocol described by Sembiring et al.^22^. The results were expressed as gallic acid equivalent (GAE) milligrams per gram. The total flavonoid content of CRE was assessed using a modified AlCl_3_ colorimetric method, as Chang et al.^23^ outlined. The estimation was based on the quercetin standard calibration curve, and the results were expressed as micrograms of quercetin equivalent (Qu) per gram of dry extract.

### NPs preparation

#### Chitosan NPs preparation

Chitosan NPs were synthesized using the ionotropic gelation method, following the procedure outlined by Manikandan et al.^24^. In summary, approximately 1 g of chitosan was dissolved in 2% acetic acid through magnetic stirring for 2 h. A sodium tripolyphosphate (TPP) at a concentration of 0.25% was prepared in distilled water. The TPP solution was gradually added drop by drop to the chitosan solution while stirring, ensuring thorough mixing. The resulting solution was left to stir for 12 h.

#### Chitosan NPs loaded with BRE: a preparation process

About 200 mg of BRE was dissolved in 10 ml DMSO. BER/CH-NPs were prepared by adding a dropwise berberine solution while stirring to a previously prepared chitosan/TPP solution after being stirred for 2 h. The final solution was left to stir for 12 h.

#### Chitosan NPs loaded with CRE: a preparation process

Briefly, 1 ml of CRE was dissolved in 10 ml DMSO, and then CRE/CH-NPs were prepared as described. A sample of each mixture of 5 ml was kept for further screening by electron microscope. The colloidal suspension was centrifuged at 14,000 rpm for 15 min by cooling centrifuge (Histman plus). The precipitate of each preparation was stored for further evaluation and application, and about 10 ml of each was frozen at – 20 °C and lyophilized for 48 h for further analysis.

The loading efficiency (LE%) of BER and CRE in the prepared nanoformulations was defined by the indirect method using the supernatants obtained during the provision of the formulations according to Kalalinia et al.^25^ by dissolving a known mass of each sample in 3 concentrations (25, 50, 100) in DMSO and distilled water respectively at room temperature. The concentration of BER and CRE loaded in the NPs was estimated in the supernatant using a spectrophotometer at a wavelength of 422 nm according to Tavade et al.^26^ and 517 nm according to Safriani et al.^27^ on a calibration graph linear regression equation and computed in the same way. The results were based on three separate analyses. Therefore, the BER and CRE contents were used to calculate the LE% in conformity with the following equation:

$$\text{LE\%}=\frac{\text{initial drug concentration}-\text{loaded drug concentration }}{\text{initial drug concentration}}\times 100$$

#### Morphological and physicochemical characterizations of the prepared NPs

##### Particle size analyses

The size of BER/CH and CRE/CH (NPs) was determined using a scanning electron microscope (SEM) model JEOL JSM-5300 operated at 15–20 keV, following the method described by Tahmasebi et al.^28^. Dynamic light scattering (DLS) was used for the measurements. To prepare the specimens for SEM analysis, a small amount of fresh BER/CH and CRE/CH NPs were fixed by immersing them immediately in a fixative solution called 4F1G (phosphate buffer solution) with a pH adjusted to 7.4. The fixation process took place at 4 °C for 3 h. After fixation, the specimens were postfixed in a 2% OsO_4_ solution in the same buffer at 4 °C for 2 h. They were then washed in the buffer and dehydrated at 4 °C using a series of ethanol with increasing concentrations. Next, the samples were dried using a critical point method, mounted on an AL-stub using carbon paste and coated with gold up to a thickness of 400 A in a sputter-coating unit (model JFC-1100 E). Finally, the surface diameter of the coated specimens was measured using the SEM.

##### Zeta potential analyses

The zeta potential (ZP) of BER/CH-NPs, CRE/CH-NPs, and CH-NPs, as well as both BER and CRE crude forms, was assessed using (Malvern Instruments Ltd. V 2.3) by electrophoretic light scattering at room temperature from the electrophoretic mobility using the capillary cell.

##### Differential scanning calorimetry (DSC) analyses

The thermal characteristics of BER/CH-NPs, CRE/CH-NPs, and CH-NPs, as well as crude forms of both BER and CRE, were examined using a Perkin-Elmer DSC 4000 device. Lyophilized samples weighing 5 mg were placed in an aluminum pan and compressed. Thermograms were recorded within the temperature range of 25–400 °C, employing a linear heating rate of 20 °C/min for the samples. This analysis aimed to determine if there were any compatibility issues within the formulation and assess the physicochemical properties of the active substances loaded in the formulations.

##### Fourier transform infrared spectroscopy (FTIR) analyses.

Lyophilized specimens were examined by (Bruker Tensor-37 FTIR spectrometer) with a wavelength range of 4000–400 cm^−1^ using the KBr pellet method.

##### X-ray diffraction (XRD) analyses

The X-ray diffraction (XRD) patterns of unprocessed samples lyophilized (NPs) of BER CRE and CH-NPs were analyzed using a Bruker D2 PHASER X-ray powder diffractometer. The XRD measurements were performed with a Ni filter and a Cu Kα radiation source (λ = 0.154 nm). The scan rate was set at 10°/min, and the XRD instrument operated at a voltage of 40 kV and a current of 30 mA.

### In vitro antioxidant activity assay

#### 2,2-diphenyl-1-picrylhydrazyl (DPPH) radical scavenging activity

The procedure was altered from the method that Brand-Williams et al. (1995) and Bondet et al. (1997) documented. This modified procedure added 0.1 ml of each sample to 3.9 ml of a DPPH solution (2,2-Diphenyl-1-picrylhydrazyl in methanol). The mixture was vigorously shaken and allowed to stand in the dark at room temperature for 1 h. The reduction in absorbance of the resulting solution against a blank was observed at 517 nm. The percentage of scavenged DPPH was then computed using an equation.

$$\text{DPPH scavenging \% }= \frac{Ao-As}{Ao}\times 100$$

In this case, Ao is the blank's absorbance and the sample's absorbance at 517 nm.

#### Assay of ferric reducing power (FRP)

Oyaizu's method^29^ was used with some modifications to determine the reducing power. The reaction involved a mixture containing 1 ml of each sample, 1 ml of 0.2 M sodium phosphate buffer (pH 6.6), and 1 ml of potassium ferricyanide K_3_Fe(CN)_6_ (1%, w/v), which was then incubated at 50 °C for 20 min. Subsequently, 1 ml of trichloroacetic acid (20%, w/v) was added to the mixture, followed by centrifugation at 5000 rpm for 15 min. The upper layer (1 ml) was combined with 0.2 ml of fresh FeCl_3_ (0.1%, w/v), and the absorbance was measured at 700 nm against a blank without Pot. Ferricyanide. A higher absorbance indicates a greater reducing power.

#### Assaying of phosphomolybdate reagent (PMA)

The total antioxidant capacity of the extracts was determined using the phosphomolybdate method, following the procedure outlined by Jayaprakasha et al.^30^ with some modifications. This method mixed 0.1 ml of the sample solution with 3 ml of phosphomolybdate reagent (0.6 M sulfuric acid, 28 mM sodium phosphate, and 4 mM ammonium molybdate). The resulting mixture was then incubated in a water bath for 30 min. After cooling to room temperature, the absorbance was measured at 695 nm against the blank.

### *Escherichia* coli and *Staphylococcus aureus*

#### Clinical examination of mastitis in farm cattle

Alexandria governorate dairy cows were assessed for clinical and subclinical bovine mastitis. Identifying clinical mastitis cases relied on historical data and systemic indicators such as general condition, rectal temperature, and appetite. A physical examination of the udder was conducted to check for swelling, warmth, and tenderness. Additionally, the appearance of the milk was observed for color alterations (yellow or blood-tinged), watery consistency, and the presence of pus flakes. Subsequently, milk samples were collected under strict hygienic conditions in sterile containers, and each was subjected to the California mastitis test (CMT) following the method described by Schalm et al.^31^. Furthermore, ten positive clinical and subclinical cases underwent bacteriological examination to isolate *S. aureus* and *E. coli*.

#### Bacterial isolation and identification

Each specimen was thoroughly blended, inoculated into Tripticase soy broth, and incubated aerobically at 37 °C overnight. Subsequently, a loopful of inoculum from the broth culture was streaked onto 5% sheep blood, mannitol salt agar, eosin methylene agar, and MacConkey agar plates. These plates were then incubated aerobically at 37 °C for 24–48 h, with growth being monitored daily. Additionally, *Staphylococcus aureus* and *E. coli* were subjected to morphological identification through Gram staining and macroscopic evaluation based on their colonial characteristics, encompassing colony size, shape, surface texture, color or pigment production, consistency (mucoid or non-mucoid), hemolytic activity on the blood agar, and type of hemolysis (α or ß or γ). Following this, biochemical identification for the isolated S. aureus and E. coli colonies adhered to the protocol outlined by Quinn et al.^32^.

#### Preparation of bacterial inoculum

The bacterial inoculum used to induce mastitis was prepared according to Suresh et al.^33^. A single *E. coli* and *S. aureus* colony from MacConkey agar and mannitol salt agar plates were inoculated into 4 ml of nutrient broth and incubated overnight at 37 °C. One ml of each broth culture was added to 9 ml of nutrient broth and incubated overnight at 37 °C. The overnight incubated broth cultures were subjected to serial fold dilution from 10-1 to 10-10 in sterile isotonic saline. From these, 100 µl of each dilution was spread plated on Mueller Hinton (MH) agar plates and incubated overnight at 37 °C. A viable count of the plates was done; if the number of colonies was too high to count, this dilution was discarded and not used for inoculation. The number of colonies was calculated using the formula: CFU/ml = number of colonies × reciprocal of dilution × 10.

### In vivo pilot test

The pilot test was conducted to confirm the most appropriate and proper, efficient concentration of the isolated bacteria (*E. coli* and *S. aureus*) for inducing acute inflammatory conditions as well as the most effective dose of the formulated NPs (BER/CH-NPs and CRE/CH-NPs) capable of relieving the induced inflammation to be utilized and to research the appropriate protocol to be followed in this investigation.

#### Pilot test for determination of bacterial concentration

Eighteen pregnant female albino rats weighing 180 ± 20 g were utilized and allocated into six groups (3 rats each) as follows: group 1 received 100 µl of physiological saline as the control, group 2 and 3 were infused with 100 µl of *E. coli* suspension containing 5.6 × 108 and 1.7 × 10^9^ CFU/ml, respectively, while group 4, 5, and 6 were infused with 100 µl of *S. aureus* suspension containing 5.3 × 10^7^, 2.1 × 10^8^, and 4.6 × 10^9^ cfu/ml, successively. On the 2nd day post-parturition, the offspring were removed, and the rats were infused as described by Brouillette and Malouin^34^. Under ketamine/xylazine anesthesia, rats were positioned on their back, and the teats and surrounding area were disinfected with 70% ethanol. Both left and right inguinal mammary glands (the fourth pair) were located, and the very near end of the teats was carefully cut with small scissors, approximately 1 mm, to prevent potential contamination by environmental microorganisms. The teat was then clamped with fine forceps, and the duct orifice was identified and gently infused into a 1 ml syringe, with the needle inserted approximately 3–4 mm deep into the teat canal. Following the infusion, the rat was placed in a filter cage on its back to allow the skin surface to dry, minimizing possible contamination^35^. Rats were euthanized 24 h after bacterial inoculation and mammary gland tissue samples were collected for histopathological examination, as per Ruifeng et al.^36^ and Chen et al.^37^.

#### Pilot test for determination of BER/CH-NPs and CRE/CH-NPs doses

Thirty-two lactating female albino rats were allocated individually into 16 groups and received different oral doses of BER/CH-NPs and CRE/CH-NPs dissolved in water once daily for 7 successive days by oral gavage as follows: groups 1, 2, 3, 4, 5, 6, 7 and 8 received oral doses of BER/CH-NPs 1, 5, 10, 15, 25, 50, 75 and 100 mg/kg B.W respectively, groups 9, 10, 11, 12, 13, 14, 15 and 16 received oral doses of CRE/CH-NPs 1, 5, 10, 15, 25, 50, 75 and 100 mg/kg B.W successively. On the 8th day, the offspring were eliminated, then after 1 h, the dams were infused with 100 uµ of *S. aureus* suspension containing 2.1 × 10^8^ CFU/ml into the duct of the inguinal MG, then 24 h later, blood samples were collected from rats were euthanized (At the end of the study, rats were terminally anesthetized with pentobarbital (80 mg/kg)). MG tissue samples were rapidly removed for histopathological examination.

#### Histopathological examination

Simultaneously to the time of collection, a small part of the MG tissues was excised for histopathological examination from all the animals’ groups, washed with cold normal saline solution, and dried by double sheet filter paper; the specimens were initially fixed in 10% neutral buffered formalin (pH 7.0) for 24 h and then transferred to 70% ethyl alcohol. Following this, the samples underwent clearing in xylene for 6 h, were placed in soft paraffin in a crucible, and were kept in an oven at 56 °C for 12 h. Subsequently, the samples were blocked in hard paraffin, and sections approximately 5 microns thick were cut. These sections were cleared in xylene, impregnated in paraffin wax, and cut into 5–7 µm thick sections. Harri’s hematoxylin and eosin (H&E) staining method was applied to the sections, as per Bancroft and Gamble (2008). Finally, the specimens were examined for structural changes under a light microscope.

### Consent to participate

All authors have contributed and agreed to participate in this paper.

## Results and discussion

### Bacteriological examination and identification

**Figure 1:**
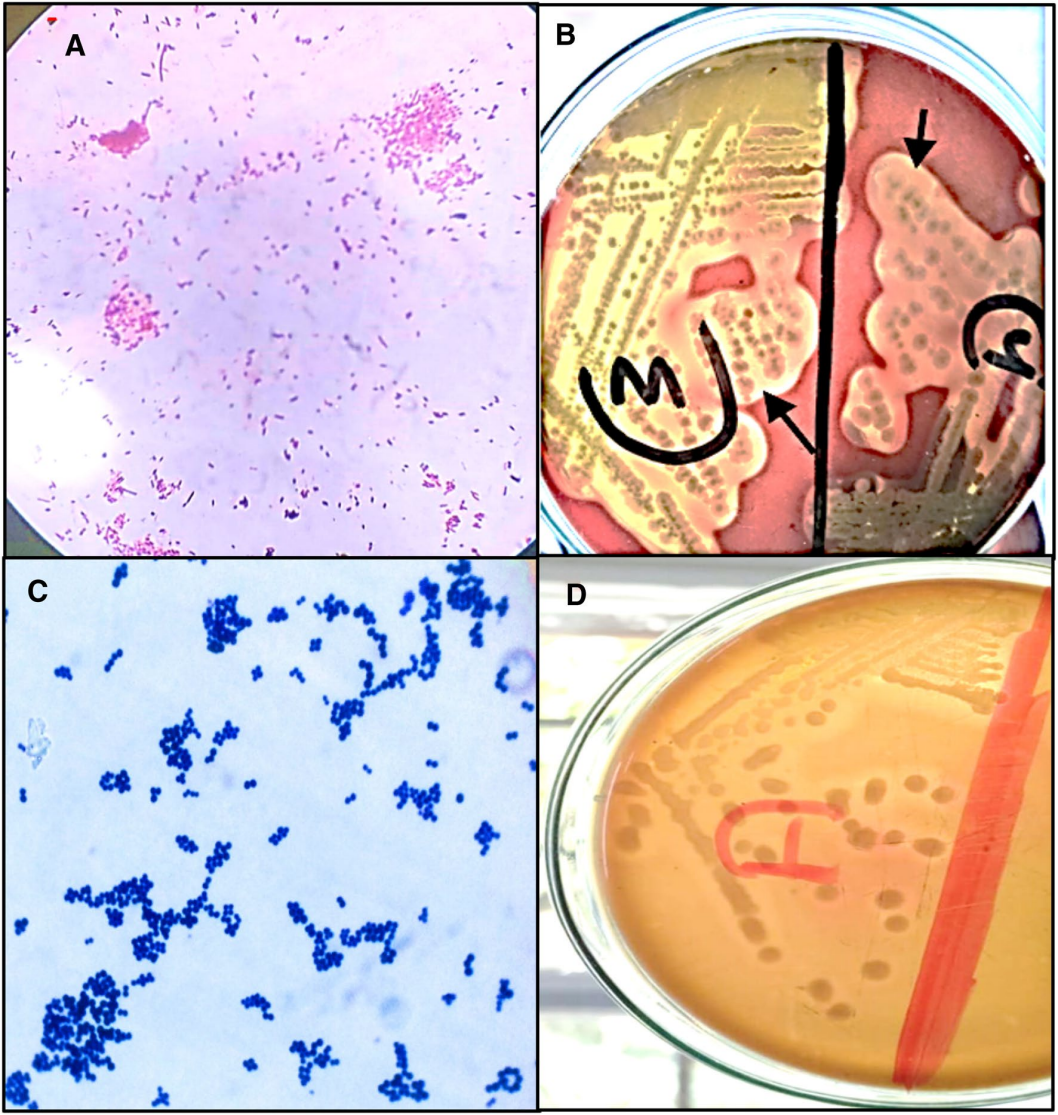
Morphological identification of isolated bacteria (*E. coli*, *S. aureus* and *St. agalactia*) from milk samples of clinical bovine mastitis cases. (**A**) *E. coli* showing Gram-negative bacilli arranged irregularly with α-hemolysis (black arrow) on 5% sheep blood agar (**B**). (**C**) *S. aureus* showing Gram-positive cocci arranged in clusters with β-hemolysis (black arrow) on 5% sheep blood agar (**D**).

Mastitis is an inflammation of the MG tissue in response to microbial infection or non-infectious causes, which leads to massive annual financial losses in the dairy industry, estimated to be about 147 $ per cow, owing to a profound alteration in milk quality and quantity^38^. Bacterial infection is the universal cause of mastitis either clinically or sub-clinically, namely due to *E. coli*, *streptococcus* spp., *Staphylococcus* spp., and others resulting in low milk yield, increased discard of raw milk, culling rate of dairy animals and the unsuitability of mastitic milk neither for human consumption nor for processing^39^. *E. coli* and *St. agalactia* from milk samples of clinical bovine mastitis cases; nonetheless, *S. aureus* was isolated from both clinical and subclinical cases, which were biochemically (Table 1) and morphologically (Fig. 1) identified. That is to say that, among 10 positive CMT milk samples, 6 cases were after clinical infection with *E. coli* (Gram-negative bacilli), *S. aureus* (Gram-positive cocci arranged in irregular clusters) and *St. agalactia* (Gram-positive cocci arranged in pairs or chains) with the ratio of 30%, 40%, and 10%, respectively, additionally to 20% subclinical *S. aureus* infected ones.

**Table 1:** Biochemical identification of isolated bacteria (*E. coli*, *S. aureus* and *St. agalactia*) from milk samples of clinical bovine mastitis cases.

Biochemical test	*E. coli*	*S. aureus*	*St. agalactea*
TSI (A/K/G/H2S)	A/G-A	A	A
Oxidase	−	−	−
Catalase	+	+	−
Indole	+	−	−
Urease	−	+	−
Mannitol	+	+	−
Motility	+	−	−

**Figure 2:**
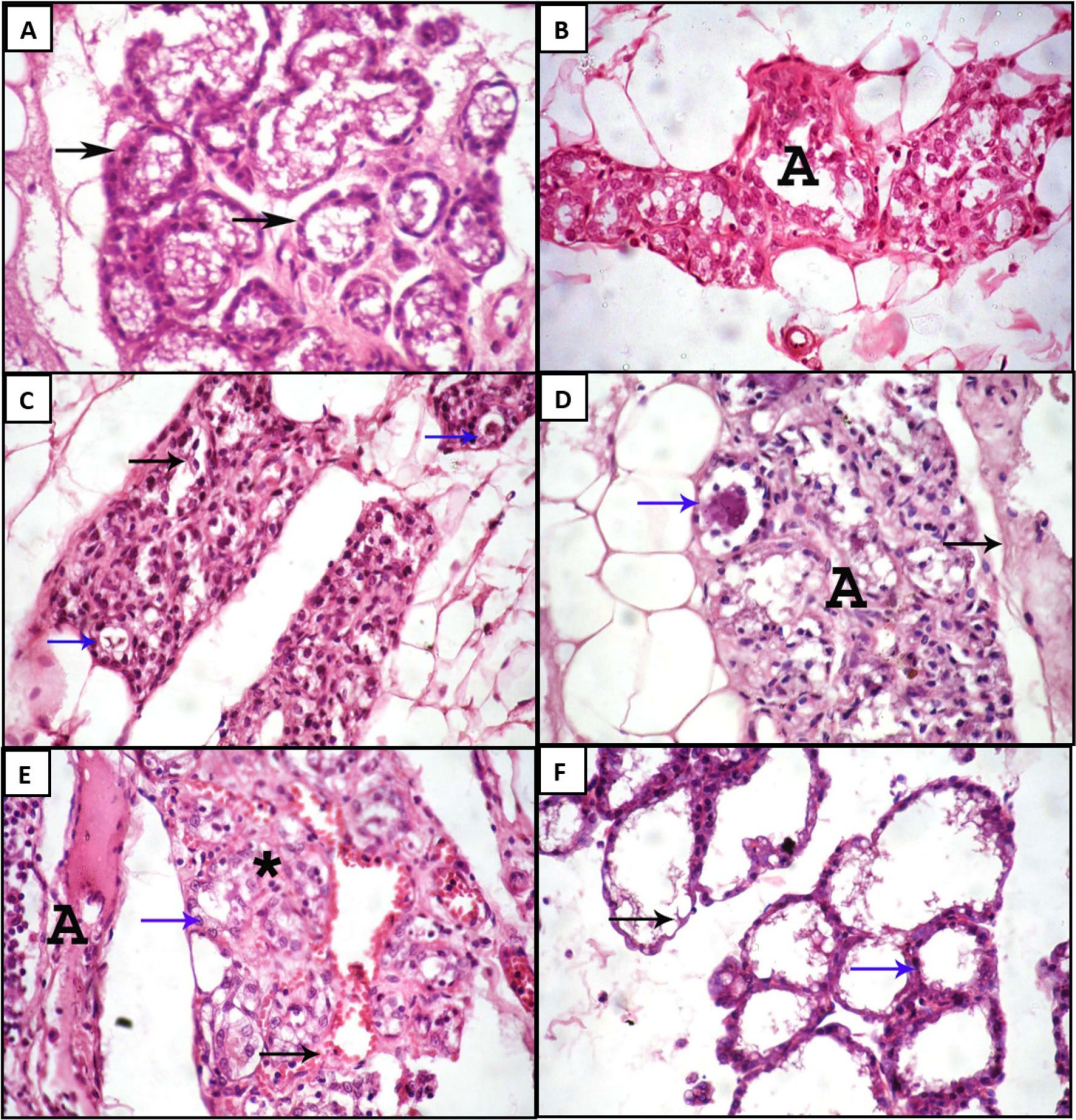
Photomicrograph of rat mammary gland tissues infected with different *E. coli* and *S. aureus* doses. (**A**) The control group showed the presence of normal architecture and details with the presence of normal intact glandular epithelium and the presence of milk secretion remnant within the alveolar lumen (arrows). (**B**) received *E. coli* (1.7 × 10^9^ CFU/ml) showed severe destruction and degeneration of alveoli. (**C**) received *E. coli* (5.6 × 10^8^ CFU/ml) showed the presence of desquamation (black arrow) and vacuolation (blue arrows) of glandular epithelium. (**D**) received *S. aureus* (4.6 × 10^9^ CFU/ml) showed the presence of degenerated alveoli, desquamated glandular epithelium within the lumen (blue arrow), and interstitial edema (black arrow). (**E**) received *S. aureus* (2.1 × 10^8^ CFU/ml) showed the presence of degenerated alveoli (asterisk) with other alveoli with vacuolated glandular epithelium (blue arrow), interstitial hemorrhage (black arrow) and interstitial edema with mononuclear inflammatory cells infiltration. (**F**) received *S. aureus* (5.3 × 10^7^ CFU/ml) showed the presence of some alveoli with degenerated glandular epithelium (black arrows) and others with intact epithelium (blue arrows). H&E. × 400.

The bacterial count used for induction of mastitis was determined according to the pilot test; the degree of histopathological destruction of the MG tissue increases with the higher bacterial count inoculated compared to the control group (Fig. 2A). Thus, the most suitable symptomatic *E. coli* and *S. aureus*-induced mastitis count was investigated as 5.6 × 10^8^ (Fig. 2C) and 2.1 × 10^8^ CFU/ml (Fig. 2E), respectively, as those who were inoculated with higher bacterial count showed more destructive histopathological tissue lesions (Fig. 2B,D), due to the high PMN cells recruited in response to higher bacterial count will phagocytose the bacterial cells to reduce their colonization which lowers the effectiveness of the administrated drugs^34^ as well as the potential damage of MG epithelial cells by respiratory burst and degranulation^40^ which could lead to the chronic inflammatory response^41^. However, the minor challenged bacterial count will result in a more significant response^42^, as shown in Fig. 2F.

Antimicrobial resistance (AMR) is one of the most essential issues, rendering the treatment of infectious diseases more challenging and impacting public health; thus, the WHO has announced its growing ineffectiveness^43^. Current therapies are becoming less effective due to AMR. For example, a study reports that antibiotics like penicillin and cephalosporins are increasingly ineffective against common pathogens such as E. coli and Klebsiella pneumoniae due to resistance mechanisms^44^. Additionally, the Rising resistance in Neisseria gonorrhoeae, complicates the treatment of gonorrhea and leads to limited therapeutic options^45^. Accordingly, in recent decades, a trend has been to intensify research concerning the medicinal application of phytochemicals as antimicrobial alternatives^46^, specifically through a nano-drug delivery system to promote their bioavailability, hydrophobicity, and absorption^47^. Recent research has highlighted phytochemicals as antimicrobial alternatives. Abreu et al.^7^ showed berberine's efficacy against resistant bacteria, while^8^ highlighted curcumin's broad-spectrum activity. Thymol and carvacrol's effectiveness. Nanocarriers enhance these phytochemicals' bioavailability^9^. Das et al.^10^ showed improved efficacy with curcumin-loaded nanoparticles, Zhang et al.^11^ increased berberine's bioavailability with liposomes This study illustrated the potential of BER and CRE-loaded chitosan nanoformulations compared to their crude form.

### CRE phytochemical screening

The total phenolics and flavonoids are the most abundant secondary metabolites representing the major antioxidants and free radical scavengers. Moreover, it has been reported that flavonoid contents within CRE have a broad spectrum of pharmacological activities, including antioxidant, antimicrobial, and antimutagenic effects^48^. The main CRE phenolic contents were denoted in (Table 2) as catechol, cinnamic, salicylic acid, pyrogallol, gallic, and vanillin. However, the principal flavonoids were rutin, apigenin, luteolin, and apegenin. Also, the CRE's total phenolic content was 59.4 mg/g, slightly exceeding the total flavonoid content (44.6 mg/g) reported previously by Siroua et al.,^49^. Thus, the previously illustrated results justify that the Egyptian CRE could contribute to its prospective medicinal properties.

**Table 2:** Total phenolic and flavonoids contents in CRE extract.

Phenolic compounds			Flavonoid compounds		
RT#	Compound	Concentration (µg/gm)	RT#	Compound	Concentration (µg/gm)
4.0	Catechol	13.22	5.2	Rutin	14.26
6.8	Cinnamic	12.75	7.0	Quersestin	7.85
8.8	Pyrogallol	4.55	9.0	Luteolin	8.36
10.0	Gallic	3.42	10.0	Apigenin	10.46
12.0	Salicylic acid	7.14	5.2	Rutin	14.26
15.0	vanillin	0.97			
Total phenolics (mg GAE/g dry extract)		54.9	Total flavonoids (mg QuE/g dry extract)		44.6

### Morphological and physicochemical characterizations of the synthesized NPs

The synthesized NPs loading efficiency percent (LE%) was 91.3% and 86.5% for BER/CH-NPs and CRE/CH-NPs, respectively. This might be due to the concentration's suitability and the inter-molecular interaction of the cross-linking reactants, including TPP^50^. That is to say, chitosan provided the loaded bioactive agents with more stability and integrity regarding the multilayer coating, which protects against the GIT environment and metabolic degradation^51^. Additionally, the positive surface charge and the mucoadhesive properties of CH-NPs allow its adherence to the mucus membrane, as well as the sustained release of the loaded drug; thus, chitosan has been appointed as an optimal drug carrier since it improved the solubility and bioavailability of nano-encapsulated material^52^.

**Figure 3:**
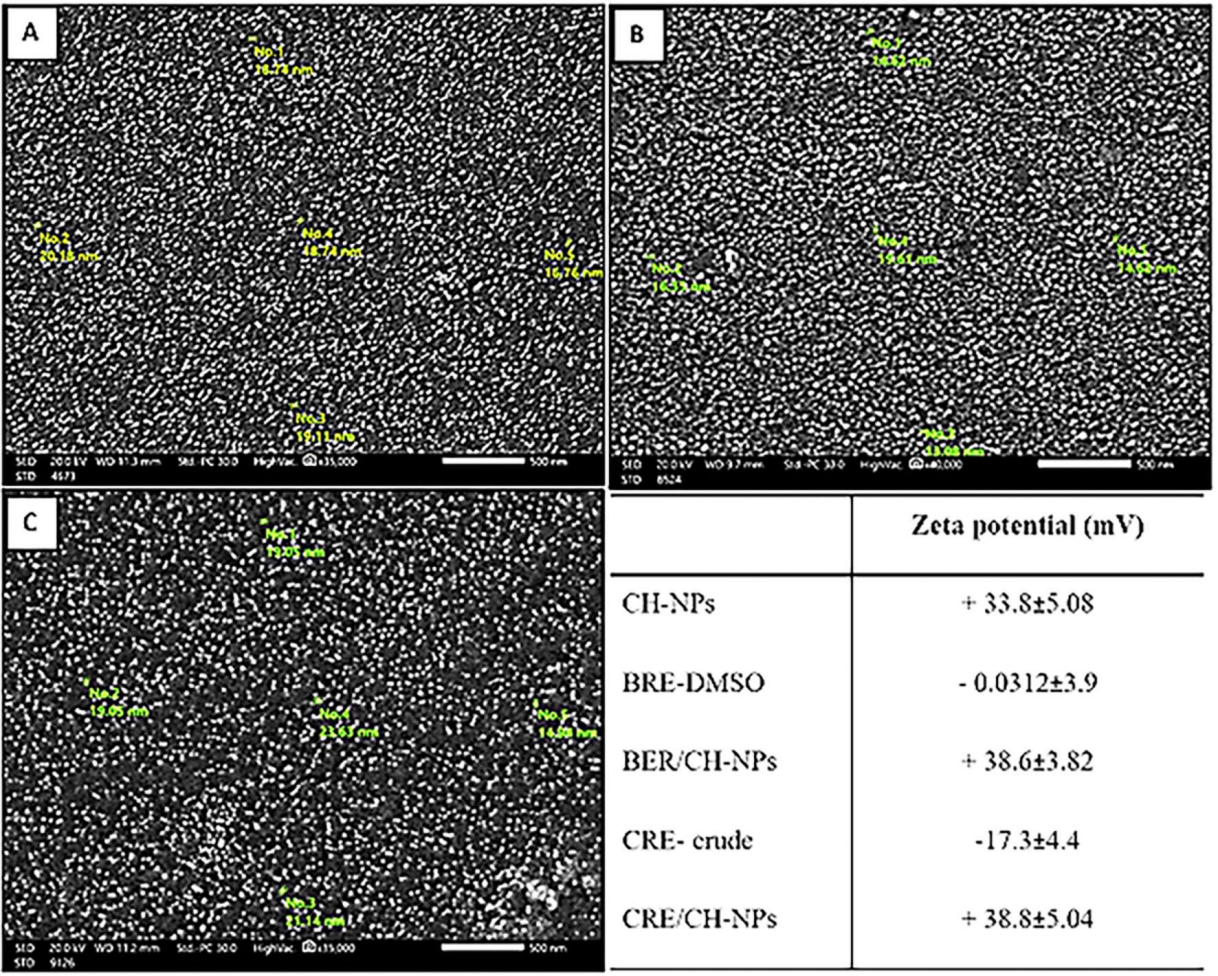
Scanning Electron Microscope (SEM) micrograph and zeta potential of nanoparticles. (**A**) SEM image of CH-NPs (Chitosan nanoparticles). (**B**) SEM image of BER/CH-NPs (Berberine-loaded chitosan nanoparticles). (**C**) SEM image of CRE/CH-NPs (*Cyperus rotundus* rhizomes-loaded chitosan nanoparticles) indicated that the particles possessed a uniform, spherical-like shape with an average diameter.

Notably, NPs with less than 200 nm particle sizes can escape renal clearance and pervade into disparate tissues^53^. The scanning micrograph of CH-NPs, BER/CH-NPs and CRE/CH-NPs by SEM denoted that the particle had a homogenous spherical-like structure with a mean diameter of 16.8 ± 2.3, 16.7 ± 2.3 and 18.3 ± 3.2 nm, respectively (Fig. 3), despite of the fact that the particles of CRE are small granular like structure^54^ and that of berberine showed an irregular prism-like crystals design with a broad particle size distribution which was responsible for their poor bioavailability and GIT absorption in contrast to the nano formulations. Similarly, the polymeric polycationic nature of chitosan enables it to create polyelectrolyte complexes by interacting with polyanions^50^, which harmonizes the results in (Fig. 3) that designated positive ZP of BER/CH-NPs and CRE/CH-NPs with ZP value 38.6 ± 3.8 and 38.8 ± 5.4 mV, respectively despite the negatively charged crude forms which have a lower value accounting for − 0.0312 ± 3.9 mV for BER-DMSO solution and − 17.3 ± 4.4 mV for CRE-DMSO, respectively because of the high value positively charged CH-NPs (+ 33.8 ± 5.08 Mv). Hence, the formulated NPs exert higher bioavailability, cellular uptake, absorption, and stability because the electrostatic repulsive forces of the particles with ZP between + 30 and + 60 mV prevent their aggregation in additionally to their increased protein-binding capacity, which strengthens the binding between the administrated NPs and the biological tissue membranes and consequently augment the cellular uptake level^55^. This came in contrast to the particles with smaller ZP values, which pursue flocculation and aggregation due to the action of the Van der Waals attraction forces, resulting in poor stability and bioavailability^56^**.** The negative zeta potential of CRE and BER crude extract may be attributed to the high negative zeta potential of DMSO as recorded by Macedo et al.^57^, which showed that the zeta potential of acetylated cashew gum dissolved in DMSO was about − 39.8 Mv.

**Figure 4:**
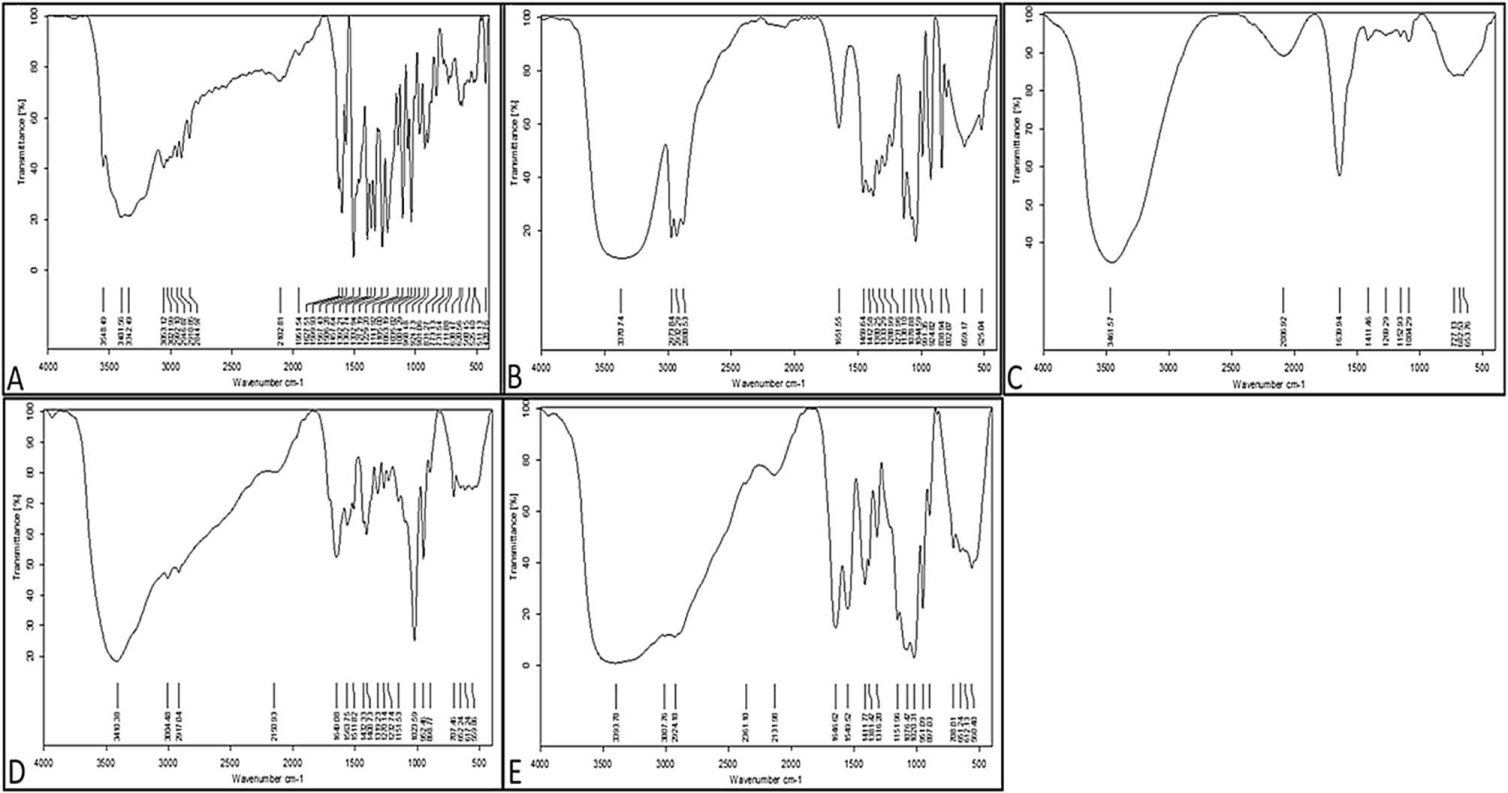
Fourier transforms infrared spectroscopy (FTIR) spectra of BER and CRE in crude and nanoparticles. (**A**) FTIR spectrum of crude BER (Berberine), indicating characteristic peaks of BER in its raw form. (**B**) FTIR spectrum of crude CRE , showing the characteristic absorption bands of the crude extract. (**C**) FTIR spectrum of CH-NPs (Chitosan nanoparticles), displaying the specific functional groups of chitosan. (**D**) FTIR spectrum of BER/CH-NPs, showing the characteristic peaks of both chitosan and berberine, confirming successful encapsulation. (**E**) FTIR spectrum of CRE/CH-NPs, illustrating the characteristic peaks of chitosan and the crude extract, indicating successful encapsulation. The FTIR spectra in each panel highlight the functional groups present in the samples, confirming the chemical composition and successful loading of BER and CRE into the chitosan nanoparticles.

FTIR for BRE, CRE, CH-NPs, BRE/CH-NPs, and CRE/CH-NPs was performed in the range of 4000–400 cm^−1^, indicating sharp peaks with appropriate intensities as the vibrational changes play a marked role in the intermolecular liaisons in solid materials to identify the molecular distribution of the phytochemicals within the chitosan NPs molecular scaffold. On one hand, (Fig. 4A) illustrates the FTIR spectra of berberine chloride, including significant peaks observed at 3401.56 cm^−1^ indicating (O–H) stretching vibration, 3053.12 cm^−1^ for the hydrophilic quaternary ammonium (N^+^) group, which has been related to its binding to 4 hydrophobic aromatic heterocyclic hydrocarbon groups via covalent (C–N) bonds, 2946.87 cm^−1^ alternative to saturated (C–H) stretching, 2844.97 cm^−1^ corresponding to (–OCH_3_) methoxy group. The 1627.51 cm^−1^ and 1599.93 cm^−1^ peaks were designated for heterocyclic amines as (C–N) bonds and (C=N^+^) quaternary iminium cation double bonds in the molecule stretching vibration band, respectively. The peaks at 1567.43 cm^−1^ were assigned for (C=C) bending vibration in the aromatic ring, 1506.28 cm^−1^ for the furyl group, and skeleton vibration of aromatic (C=C) ring stretching vibrations, while that at 1457.64 cm^−1^ identified (–CH_2_) methylene and those at 1392.21 cm^−1^ and 1362.74 cm^−1^ devoted (–CH_3_) stretching. The peaks at 1332.94–1035.02 cm^−1^ conferred to (C–O–C) bonding, whereas 1105 cm^−1^ to (C–O) stretching and deformation of the aromatic ring, those at 968.48–731.54 cm^−1^ to (=C–H) out of the plane and a halide band for chloride was allocated at 620.56–560.56 cm^−1^. This result coincided with previous studies^18^. On the other hand, the peaks represented in Fig. 4B identified the existence of the main constituents of CRE., notably, a broad band at 3370.74 cm^−1^ (–OH), 2973.84–2880.53 cm^−1^ (C–H stretching), 1651.55 cm^−1^ (C=O), 1333.29 cm^−1^ (phenolic-OH), 1238.10 cm^−1^ (–C–O–C), 1078.88 cm^−1^ (C–O) and 1044.59 cm^−1^ (C–N) confirmed that extract possess different chemical compounds as reported by Kakarla et al.^54^, who recorded a different wavelength and shift patterns on FTIR analysis of CRE. Also, the result was in parallel with^58,59^**.** Admittedly, CH-NPs were formed by ionic gelation between TPP and free chitosan thus the cross-linking between the chitosan chains transferred some amide group-related peaks as expressed in Fig. 4C by a peak at 1639.94 cm^−1^ for amide I vibration (–NH_2_ bending) and (C=O) stretching in the amide group, bands at 1459.64 cm^−1^ (amide II) implying for the electrostatic interaction between NH^3+^ of chitosan and the phosphate group of TPP, 1084.29 cm^−1^ conferred to PO_4_^–2^ and C–O stretching, 1411.46 cm^−1^ (–CH_2_ wagging) and a strong peak at 3461 cm^−1^ assigning for (–OH) which is the same result with Ma et al.,^60^, Lustriane et al.,^61^. Moreover, the encapsulation of BER and CRE into CH –NPs by the lack of the characteristic peak of methoxy group in BER/CH-NPs and the presence of weak distinguishing peaks of amide bonds at 1408.73 cm^−1^ and 1319.23 cm^−1^, which could be accorded to the condensation reaction between the carbonyl group of BER and the amino group of CH-NPs or an addition reaction with (–OH) group presented by a broad peak at 3410.38–3004.48 cm^−1^ (Fig. 4D,E), suggesting that the previous functional groups participated in the cross-linking reaction of BER/CH-NPs^62^. Likewise, the alcohol/ phenol (–OH) stretch shifted to 3393 cm^−1^, and the aromatic asymmetric band to 1549.52 cm^−1^ in CRE/CH-NPs^54^.

**Figure 5:**
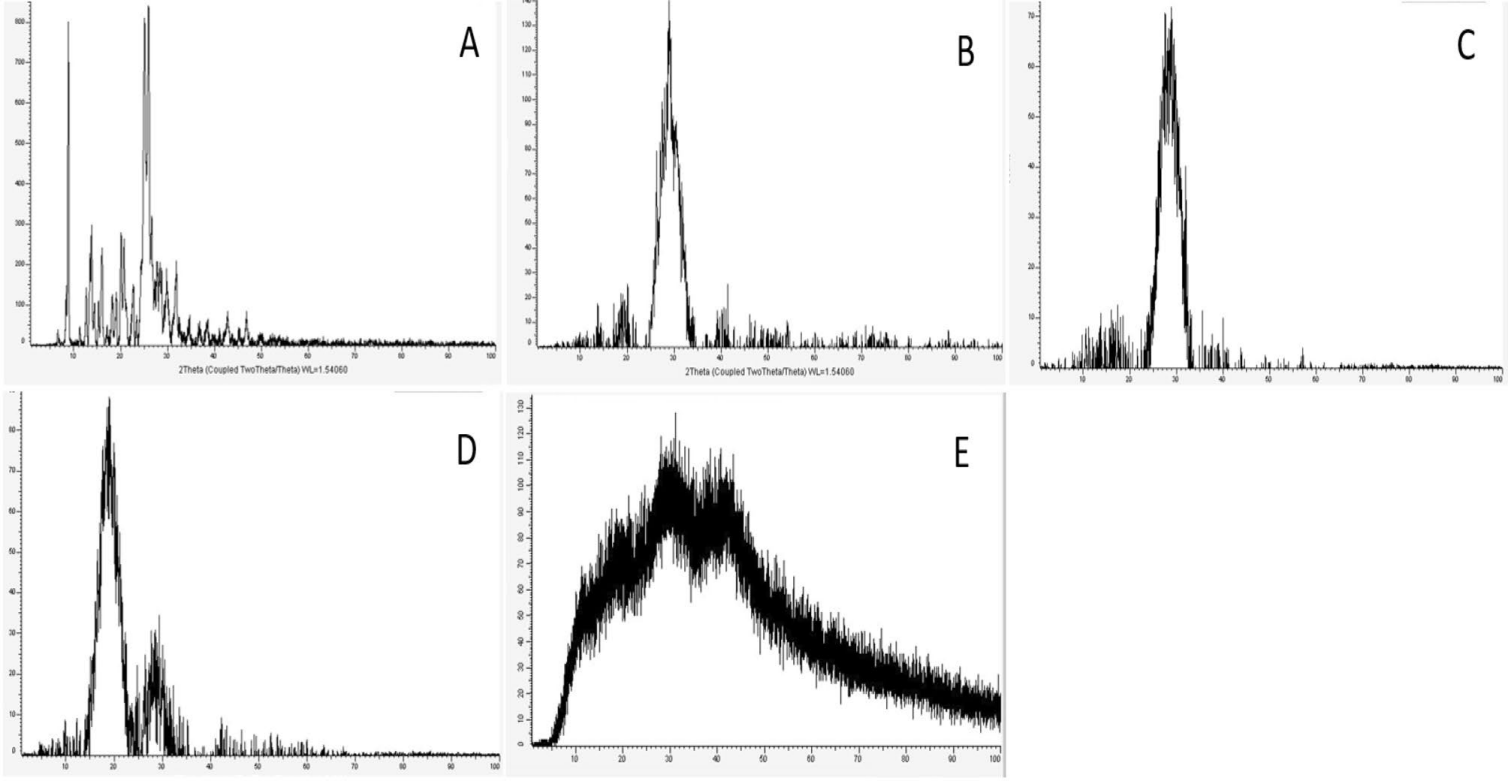
X-Ray Diffraction (XRD) patterns of BER and CRE in crude and nanoparticles. (**A**) Crude BER, showing crystalline peaks. (**B**) Crude CRE, displaying distinct crystalline peaks. (**C**) CH-NPs, indicating an amorphous structure. (**D**) BER/CH-NPs, showing peaks of both berberine and chitosan, confirming encapsulation. (**E**) CRE/CH-NPs, combining peaks of crude extract and chitosan, confirming encapsulation. These patterns highlight the structural integrity and successful loading of the compounds into nanoparticles.

Furthermore, the physical existing status of the studied materials was confirmed by a comparative XRD spectrum in the 2θ range of 0°–100° at a scan speed of 10°/min for CH-NPs, BER, and CRE in both crude and nano-formulated modalities recorded in (Fig. 5). CH-NPs diffractogram showed a low-intensity diffraction peak at 2θ of 27.68° at a diffraction plane 422. The crystalline property of crude BER was identified by two intense sharp peaks at 2θ of 8.94° and 26.04°^63,64^. Likewise, CRE by a sharp peak at 29.07° which disappeared in the diffraction peaks of their corresponding nano-formulations and was replaced by a sharp broad peak and another small one at 2θ of 20.26° and 28.32° for BER/CH-NPs; besides, a wide one at 31.2° for CRE/CH-NPs with less intensity for both suggesting a successful encapsulation within CH-NPs^63^ which could be assigned for the planar staking of amorphous polymer chains formed by the copolymerization of the nano formulated materials due to the formation of a complex between CH, BER, and CRE individually through the interaction among (–OH), carbonyl and (–NH) groups as stated by FTIR and the formation of cross-link network rendering the NPs more stable^65^. Consequently, less amorphous or crystalline forms of drugs could be more simply solubilized and had improved dissolution rates compared to their corresponding crystalline analog^66^.

**Figure 6:**
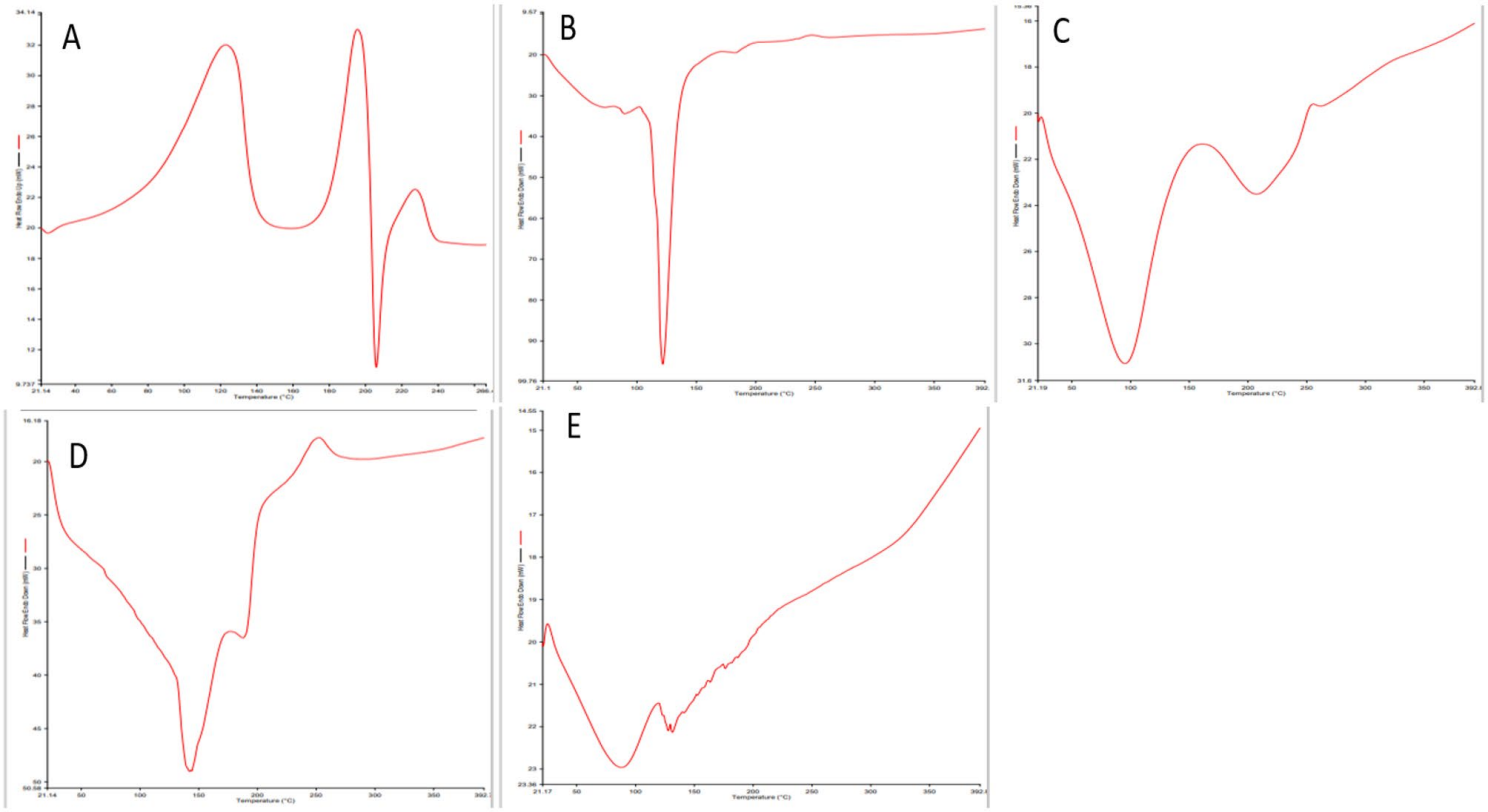
Differential Scanning Calorimetry (DSC). Thermal characteristics of BER/CH-NPs, CRE/CH-NPs, and CH-NPs of BER and CRE in crude and nanoparticles. (**A**) crude BER, (**B**) crude CRE, (**C**) CH-NPs, (**D**) BER/CH-NPs, (**E**) CRE/CH-NPs. The thermogram of berberine chloride shows peaks at 123.12 °C, 162.35 °C, and 205.86 °C, indicating crystallinity. CRE has a peak at 121.79 °C, also indicating crystallinity. CH-NPs show a broad peak at 95.50 °C. BER/CH-NPs and CRE/CH-NPs show broader peaks at 142.10 °C and 90.30 °C, indicating polymer interaction and homogeneous encapsulation with lower crystallinity and higher hydrophobicity than the crude forms.

Moreover, the thermal behavior and stability of the studied materials were investigated by performing DSC analysis^67^; as shown in Fig. 6, the diminished enthalpy value is representative of reduced crystallization due to decreased particle size^68^. The thermogram of berberine chloride illustrated a pair of exothermic peaks at 123.12 °C and 162.35 °C as well as a sharp narrow endothermic peak at 205.86 °C^69^, while CRE showed a sharp endothermic peak at 121.79 °C; indicating their crystalline nature. The DSC data of CH-NPs revealed a broad endothermic peak at 95.50 °C^70^. Nevertheless, BER/CH-NPs and CRE/CH-NPs showed no characteristic peaks of the crude forms. They shifted to broader endothermic peaks at 142.10 °C and 90.30 °C, respectively, shifting toward that of CH-NPs, which revealed the interaction between both polymers with the formation of polymer complexes^70^ and their homogenous encapsulation within CH-NPs with no incompatibility with lower crystallinity and higher hydrophobicity than the crude forms^50^.

### In vitro antioxidant activity of BER/CH-NPs and CRE/CH-NPs

Methods for direct evaluation of the antioxidant activity of bioactive compounds of herbal origin due to their comprehensive complex composition and chemical diversity, most commonly DPPH, FRP, and PMA; besides, chitosan-loaded NPs of plant origin had been found to have a significantly enhanced antioxidant activity^71^. Results in Table 3 illustrated that the antioxidant activity of Nano formulated particles soared substantially in a concentration-dependent manner compared to their crude form following previous literature. For instance**,** MIR et al.^72^ pointed out the increased DPPH radical scavenging activity of BER/CH-NPs, which could be accounted for its expanded solubility, dissolution, and stability as DPPH is a proton acceptor that served as a free radical. Typical of this, Younis et al.^53^ suggested that the advantage of BER as an unrestrained radical scavenger alkaloid as well as its overall physicochemical properties such as solubility, hydrophobicity, dissolution rate, and releasing profile with a subsequent reinforced pharmacokinetics, pharmacodynamics, distribution, and therapeutic potential through a more enhanced FRP and PMA of BER encapsulated in bovine serum albumin NPs than pure BER. First, FRP is based on an electron transfer reaction, which produces Perl’s Prussian blue color due to the reduction of Fe^3+^ to Fe^2+^ by a potentially reducing substance^73^. Secondly, PMA has been used to determine the total antioxidant activity via the decrease in molybdate (VI) to molybdate (V) complex with a distinguished bluish-green color^74^. As such, Kakarla et al.^54^ documented that CRE/CH-NPs exhibited more potent antioxidant activity than CRE and BER/CH-NPs, which could be regarded as prosperous phenolic contents. Equally, the worthiness of chitosan to boost the antioxidant activity of a loaded material has been attested by Bhoopathy et al.^73^ and Hadidi et al.^75^, who studied the antioxidant behavior of curcumin and clove essential oil, respectively formulated as chitosan loaded NPs.

**Table 3:** In vitro antioxidant activity of berberine, cyperus extract, BER/CH-NPs and CRE/CH-NPs.

	DPPH %	PMA (mg/g)	FRP (mg/g)
CH-NPs	26.3 ± 1.2^e^	8.5 ± 0.5^e^	11.1 ± 0.1^e^
Bereberine	53.6 ± 3.5^d^	41.5 ± 0.8^d^	27.6 ± 0.4^d^
BER/CH-NPs	69.2 ± 1.1^c^	49.2 ± 0.3^c^	38.3 ± 0.2^c^
Cyperus extract	74.3 ± 2.6^c^	47.6 ± 1.2^c^	39.8 ± 0.8^c^
CRE/CH-NPs	88.5 ± 1.3^b^	65.2 ± 0.3^b^	47.5 ± 0.3^b^
BER + CRE/CH-NPs	98.2 ± 1.1^a^	76.2 ± 0.4^a^	65.3 ± 0.2^a^

Values are expressed as means ± SE. Mean values within the same column with different superscript letters are a statistically significant difference at p ≤ 0.05.

### Anti-inflammatory concentration of BER/CH-NPs and CRE/CH-NPs

Previous studies have found that *S. aureus* is the most prevalent pathogen causing both clinical and subclinical mastitis with higher incidence to induce a chronic infection in comparison to *E. coli*, resulting in a devastating loss in milk production and with less than half of treated animals were responsive to administrated drugs^76^. Thus, in this study, an isolated *S. aureus* strain from a clinical bovine mastitis case discussed above was used to induce mastitis in rats, and multiple trials of different prophylactic doses of BER/CH-NPs and CRE/CH-NPs were carried out.

**Figure 7:**
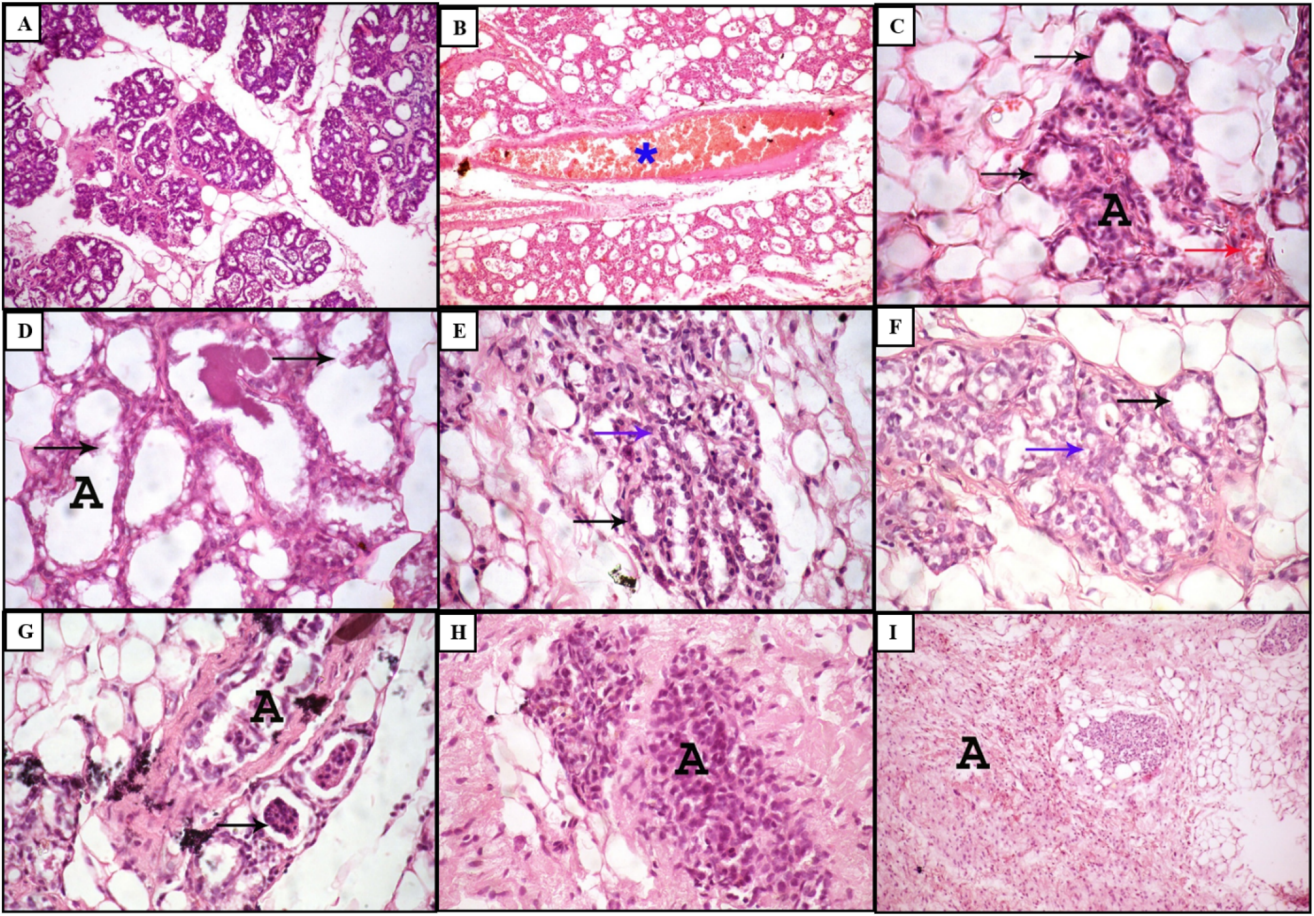
Photomicrograph of rat mammary gland tissues treated with different doses of BER/CH-NPs. (**A**) control group showing the presence of mammary lobules containing alveoli filled with milk secretion with normal histo-architecture. (**B**) 1 mg/kg BER/CH-NPs group shows the presence of blood vessel congestion (asterisk). (**C**) 5 mg/kg BER/CH-NPs group shows some milk alveoli with normal histo-architecture (black arrows), with some degenerated alveoli with minimal congestion of blood vessels (red arrow). (**D**) 10 mg/kg BER/CH-NPs group shows some enlarged alveoli due to alveolar boundary destructions (black arrows). (**E**) The 15 mg/kg BER/CH-NPs group showed the presence of some alveoli with apparent histo-architecture (black arrow) and many degenerated alveoli (blue arrow). (**F**) 20 mg/kg BER/CH-NPs group shows some alveoli with vacuolar degeneration of epithelium (black arrow) and some degenerated alveoli (blue arrow). (**G**) The 50 mg/kg BER/CH-NPs group shows epithelial cell debris within the alveolar (arrow) and ductular lumen. (**H**) 75 mg/kg BER/CH-NPs group shows complete alveolar degeneration and necrosis with mononuclear inflammatory cell infiltration. (**I**) The 100 mg/kg BER/CH-NPs group shows the presence of widely distributed interstitial mononuclear inflammatory cell infiltration (**A**). H&E. (× 100).

**Figure 8:**
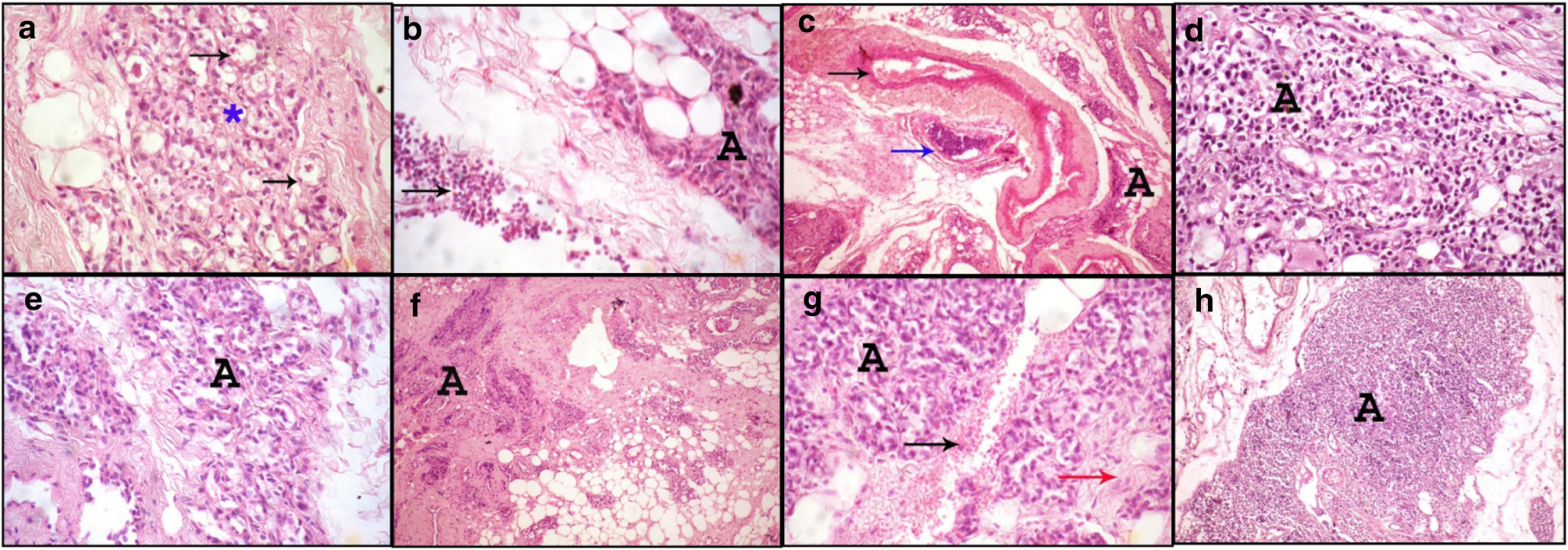
Photomicrograph of rat mammary gland tissues treated with different doses of CRE/CH-NPs. (**a**) 1 mg/kg CRE/CH-NPs group shows the presence of vacuolation of the alveolar epithelium (black arrows) with some degenerated alveoli (asterisk). (**b**) 5 mg/kg CRE/CH-NPs group shows minimal alveolar degeneration (A) with interstitial inflammatory cell infiltration. (**c**) 10 mg/kg CRE/CH-NPs group showing the presence of congestion of blood vessel (black arrow) and presence of blood vessel engorged with mononuclear inflammatory cells (blue arrow) and area of degenerated alveoli with mononuclear inflammatory cells infiltration (A). H&E. (× 100). (**d**) The 15 mg/kg, CRE/CH-NPs group, shows widely distributed alveolar degeneration and necrosis with mononuclear inflammatory cell infiltration (A). (**e**) 25 mg/kg CRE/CH-NPs group showed a wide area of degenerated milk alveoli with fibrosis (A). (**f**) 50 mg/kg CRE/CH-NPs group shows a large area of degenerated alveoli with massive mononuclear inflammatory cell infiltration (A) H&E. (× 100). (**g**) 75 mg/kg, CRE/CH-NPs group, showing the presence of a wide area of degenerated milk alveoli (A) with hemorrhage (black arrow) and fibrosis (red arrow) H&E. (× 400). (**h**) 100 mg/kg CRE/CH-NPs group showing the diffused area of alveolar necrosis with a massive number of mononuclear inflammatory cells infiltration H&E. (× 100).

Concerning histopathological results for determination of the preventive doses of both BER/CH-NPs and CRE/CH-NPs before inducing mastitis by intra-mammary inoculation of 100 µl of 2.1 × 10^8^ CFU/ml *S. aureus*, light microscopy examination revealed that MG of control group exhibited normal histo-architecture of mammary lobules as the alveoli appeared with intact glandular epithelium (Fig. 7A). Regarding the treated groups in comparison with the induced mastitis group with 5.3 × 10^7^ CFU/ml *S. aureus* mentioned above, MG tissue of BER/CH-NPs 1 mg showed moderate congestion of blood vessels (Fig. 7B), while mammary tissues of BER 5mg showed the presence of the most of milk alveoli with normal histo-architecture. At the same time, some of them appeared to have degenerated alveoli with minimal congestion of blood vessels (Fig. 7C). On the other hand, the mammary gland sections of BER/CH-NPs 10 mg revealed the presence of distended alveoli due to the rupture of boundaries between them (Fig. 7D). Mammary tissue sections of the BER/CH-NPs 15 mg maintained some alveoli of normal architecture. Still, most of the alveoli appeared with degenerative changes (Fig. 7E), and the same changes appeared during the examination of mammary tissues in the BER/CH-NPs 25 mg group (Fig. 7F). The desquamated alveolar epithelium was accumulated in alveoli and mammary ductules in the BER/CH-NPs 50 mg group (Fig. 7G). Alveolar necrosis was evoked in the BER/CH-NPs 75 mg group (Fig. 7H), and widely distributed mononuclear inflammatory cell infiltration in interstitial tissues was obvious in the BER/CH-NPs 100 mg group (Fig. 7I). Regarding the histopathological findings of CRE/CH-NPs, mammary tissue alveoli epithelium appeared vacuolated or degenerated in the CRE/CH-NPs 1 mg group (Fig. 8a). This degeneration increased in severity with the appearance of minimal interstitial mononuclear inflammatory cell infiltration in the CRE/CH-NPs 5 mg group (Fig. 8b). Several focal dispersed areas of alveolar degeneration with severe congestion of blood vessels and their engorgement with inflammatory cells were recorded in the CRE/CH-NPs 10 mg group (Fig. 8c). A wide distribution of alveolar degeneration and necrosis with mononuclear inflammatory cell infiltration was prominent in the CRE/CH-NPs 15 mg group (Fig. 8d), and fibrosis was detected in the CRE/CH-NPs 25 mg group (Fig. 8e). Massive alveolar degeneration and mononuclear inflammatory cell infiltration were clear in the CRE/CH-NPs 50 mg group (Fig. 8f), and hemorrhage with fibrosis accompanied alveolar degeneration in the CRE/CH-NPs 75 mg group (Fig. 8g). Diffused areas of alveolar necrosis with many mononuclear inflammatory cell infiltrations appeared in mammary sections of the CRE/CH-NPs 100 mg group (Fig. 8h). Therefore, the most acceptable preventive dose for BER/CH-NPs and CRE/CH-NPs is 1 mg/kg day after day, as the other doses caused more destruction to the MG tissue than the previously chosen *S. aureus*-induced mastitis group. However, the achieved sequels are moderately imperfect. This could be attributed to the ability of small-size NPs to penetrate different tissues easily through various routes, including orally, with subsequent cytotoxic reactions, particularly inflammation and oxidative stress with consequent damage to cellular components^77^. The NPs size is inversely proportional to the ratio between the surface area, volume, and biological and chemical reactivity. Hence, the number of surface molecules would be 10–50% when the NPs size ranges from 3 to 30 nm. Subsequently, the interaction between NPs surface and cellular components causes the recruitment of inflammatory cells such as neutrophils, which mediate phagocytosis reaction resulting in the production of ROS additionally to its generation through enzymatic metabolism of cytochrome P450 favorable for the initiation of direct and indirect redox imbalance, inducing a dramatic cytotoxic effects^78^. For emphasis, the data represented in Table 4 expressed that the pro-inflammatory cytokines (TNF-α, IL-4, IL-6, IL-18), MPO concentration, ALT activity, and creatinine concentration were significantly risen in S. aureus-induced mastitis in comparison to the control group, however, their levels climbed gradually in the treated groups especially in BER/CH-NPs 1mg and CRE/CH-NPs 1mg groups compared to the mastitis group. This could be clarified by the fact that *S. aureus*-induced mastitis resulted in the recruitment of neutrophils into the MG with subsequent up-surged MPO concentration^33^ for the merit of killing the invading bacteria through the process of respiratory burst. Besides, the concentration of MPO is also parallel to the oxidative stress level and inflammatory cascades' activity as they increase vascular permeability and the influx of additional PMN^79^. The bacterial infection complemented by the therapeutic intervention with high doses of the formulated NPs resulted in extravagant activation of immune cells followed by a systemic dysregulated inflammatory reaction known as cytokine storm accompanied by an excessive generation of pro-inflammatory cytokines followed by tremendous tissue damage and organ dysfunction with symptoms of anorexia, fatigue, myalgia. It could be lethal^80^, which harmonized the elevated indices of liver and kidney function demonstrated in Table 3, as well as the anti-mortal manifestations of the experimental animals treated with high doses in comparison to those treated with lower ones.

**Table 4:** Effect of different doses of BER/CH-NPs and CRE/CH-NPs on pro-inflammatory cytokines, liver and kidney function on *S. aureus*-induced mastitis in rats.

	TNF-α (pg/ml)	IL-6	IL-18	IL-4	MPO	ALT (U/L)	Creatinine (mg/dl)
Control	18.7 ± 0.6^d^	27.2 ± 1.6f.	19.4 ± 6.9^d^	383.3 ± 13.2^e^	78.4 ± 7.6^e^	21 ± 2.11^d^	0.76 ± 0.03^c^
Mastitis	62.6 ± 2.1^a^	122.4 ± 9.8^a^	64.7 ± 7.6^b^	769.4 ± 21.4^b^	273.4 ± 9.1^a^	60 ± 2.65^b^	0.99 ± 0.05^a^
BER/CH-NPs 1 mg	22.1 ± 1.3^d^	33.4 ± 1.3^e^	21.7 ± 1.4^d^	432.9 ± 17.4^d^	87.1 ± 6.2^e^	27.6 ± 1.5^d^	0.79 ± 0.02^c^
BER/CH-NPs 5 mg	29.8 ± 1.4^c^	39.7 ± 1.2^e^	26.8 ± 1.3^cd^	487.3 ± 16.5^d^	103.9 ± 9.3^de^	29.7 ± 1.3^c^	0.82 ± 0.04^c^
BER/CH-NPs 10 mg	32.7 ± 1.6^c^	43.8 ± 1.4^d^	28.4 ± 1.2^cd^	541.2 ± 18.7^c^	122.7 ± 8.7^d^	37.3 ± 1.2^c^	0.87 ± 0.03^b^
BER/CH-NPs 15 mg	38.6 ± 1.4^b^	51.7 ± 2.1^c^	31.2 ± 1.6^c^	578.6 ± 21.1^c^	143.6 ± 7.8^c^	38.4 ± 1.4^c^	0.88 ± 0.04^b^
BER/CH-NPs 25 mg	44.2 ± 1.3^b^	68.7 ± 3.4^b^	37.4 ± 2.3^c^	654.1 ± 13.5^c^	157.6 ± 6.3^c^	42 ± 1.63^c^	0.91 ± 0.01^b^
BER/CH-NPs 50 mg	47.4 ± 1.2^b^	74.7 ± 8.9^b^	51.7 ± 7.5^b^	726.3 ± 19.8^b^	159.4 ± 4.9^c^	53 ± 2.11^b^	0.98 ± 0.02^a^
BER/CH-NPs 75 mg	50.9 ± 2.1^b^	92.8 ± 9.2^b^	73.2 ± 9.7^a^	790.1 ± 22.1^b^	177.1 ± 8.2^c^	56 ± 2.51^b^	0.94 ± 0.03^b^
BER/CH-NPs 100 mg	51.1 ± 0.9^b^	105.6 ± 9.7^ab^	82.8 ± 8.7^a^	909.6 ± 23.9^a^	190.6 ± 7.6^b^	90 ± 3.22^a^	1.09 ± 0.04^a^
CRE/CH-NPs 1 mg	28.6 ± 2.1^c^	42.9 ± 1.7^d^	34.7 ± 2.1^c^	421.6 ± 21.3^d^	89.3 ± 9.8^e^	24.7 ± 0.9^d^	0.75 ± 0.04^c^
CRE/CH-NPs 5 mg	37.4 ± 1.7^b^	48.1 ± 1.3^d^	42.7 ± 2.3^c^	473.8 ± 19.8^d^	101.7 ± 9.7^de^	27.9 ± 1.1^c^	0.79 ± 0.03^c^
CRE/CH-NPs 10 mg	41.7 ± 1.5^b^	55.9 ± 1.6^c^	49.8 ± 2.4^b^	537.4 ± 19.6^c^	119.6 ± 7.2^d^	31.7 ± 1.4^c^	0.87 ± 0.04^b^
CRE/CH-NPs 15 mg	44.8 ± 1.2^b^	62.3 ± 1.7^b^	54.3 ± 2.1^b^	594.6 ± 20.1^c^	149.8 ± 8.7^c^	32.4 ± 1.7^c^	0.91 ± 0.03^b^
CRE/CH-NPs 25 mg	56.2 ± 1.5^a^	73.6 ± 7.4^b^	60.7 ± 7.4^b^	640.2 ± 16.7^c^	168.6 ± 4.6^c^	35 ± 2.68^c^	0.92 ± 0.02^b^
CRE/CH-NPs 50 mg	59.3 ± 1.3^a^	87.6 ± 6.8^b^	60.7 ± 3.4^b^	771.3 ± 16.7^b^	181.7 ± 9.1^b^	59 ± 2.45^b^	1.06 ± 0.03^a^
CRE/CH-NPs 75 mg	59.4 ± 1.2^a^	115.6 ± 11.2^a^	81.5 ± 7.6^a^	845.6 ± 19.8^b^	188.5 ± 8.4^b^	79 ± 3.41^b^	1.04 ± 0.03^a^
CRE/CH-NPs 100 mg	59.7 ± 1.4^a^	125.4 ± 12.1^a^	86.7 ± 7.1^a^	828.7 ± 21.4^b^	200.9 ± 11.2^b^	95 ± 3.84^a^	1.08 ± 0.04^a^

Values are expressed as means ± SE. Mean values within the same column with different superscript letter are a statistically significant difference at p ≤ 0.05.

Generally, TNF-α is a principal regulatory pleiotropic cytokine of inflammatory responses. It functions as a pro-inflammatory cytokine and as a ligand for TNFR1 and initiates downstream signaling to induce the release of more cytokines^81^. Similarly, Liu et al.^82^ and^83^ reported the upregulation of IL-18 and IL-4 in response to S. aureus-induced inflammation. In the same way, the localized pro-inflammatory implication teased critical oxidative stress within the affected MG with further permanent tissue damage^84^. Increasing doses of NPs worsen the inflammatory reaction induced by S. aureus in a dose-dependent manner, indicating that the protective doses must be lower than 1 mg/kg body weight.

## Conclusion

To sum up, our survey implied abundant phenolic and flavonoid contents with excellent antioxidant activity. The outcomes from the characterization techniques signified the effective loading of BER and CRE into CH-NPs with a consequent enhanced hydrophobicity, stability, and bioavailability. However, the in-vivo application of BER/CH-NPs and CRE/CH-NPs before inducing *S. aureus* mastitis showed that the best preventive dose should be lower than the minimal one. Our findings suggested that oral administration of BER/CH-NPs and CRE/CH-NPs might be a prospective approach for controlling staphylococcal mastitis.

### Study limitations and future directions

Despite promising results with BER and CRE nanoparticles, this study has limitations such as small in vivo trial scales, focus on acute administration without long-term data, and partially explored nanoparticle-biological interactions. Economic and scalability issues were also not addressed. Future research should expand trial scopes, explore nanoparticle-biological interactions, extend durations, and assess economic and practical scalability aspects. Field trials and regulatory compliance are also necessary to move from lab to practical application, ensuring the feasibility and safety of these nanoparticles in the dairy industry.

## Data Availability

The datasets supporting the conclusions of this article are included within the article, and the original data of this study are available from the corresponding author upon reasonable request. The supporting data for this article are contained within the article itself, and the original study data can be provided by the corresponding author upon request.
